# Dose-Dependent Effect of Granulocyte Transfusions in Hematological Patients with Febrile Neutropenia

**DOI:** 10.1371/journal.pone.0159569

**Published:** 2016-08-03

**Authors:** Luciana Teofili, Caterina Giovanna Valentini, Roberta Di Blasi, Nicoletta Orlando, Luana Fianchi, Gina Zini, Simona Sica, Valerio De Stefano, Livio Pagano

**Affiliations:** Institute of Hematology, Catholic University, Rome, Italy; The Hospital for Sick Children and The University of Toronto, CANADA

## Abstract

It is still under debate whether granulocyte transfusions (GTs) substantially increase survival in patients with febrile neutropenia. We retrospectively examined data relative to 96 patients with hematological malignancies receiving 491 GTs during 114 infectious episodes (IE). Patients were grouped according to the median doses of granulocytes transfused during the infectious episode (low-dose group: <1.5-x10^8^ cells/Kg; standard-dose group: 1.5–3.0x10^8^ cells/Kg and high-dose group: >3.0x10^8^ cells/Kg). The impact of clinical, microbiological and GT-related variables on the infection-related mortality (IRM) was investigated. The IRM was not influenced by the number of GTs or by the total amount of granulocytes received, whereas a dose-related effect of the median dose received for IE was detected at univariate analysis (IRM of 18.4% in the standard-dose group, 44.4% in the low-dose group and 48.4% in the high-dose group, p = 0.040) and confirmed at multivariate analysis (OR 3.7, IC 95% 1.5–8.9; 0.004 for patients not receiving standard doses of GTs). Moreover, patients receiving GTs at doses lower or greater than standard had increased risk for subsequent ICU admission and reduced overall survival. The dose-related effect of GTs was confirmed in bacterial but not in fungal infections. Preliminary findings obtained from a subgroup of patients candidate to GTs revealed that levels of inflammatory response mediators increase in a dose-related manner after GTs, providing a possible explanation for the detrimental effect exerted by high-dose transfusions. GTs can constitute a valuable tool to improve the outcome of infections in neutropenic patients, provided that adequate recipient-tailored doses are supplied. Further investigations of the immunomodulatory effects of GTs are recommended.

## Introduction

Patients with cancer face prolonged periods of neutropenia. The risk of febrile neutropenia (FN) is particularly high in patients with hematological malignancies, especially in those older than 60 years [1]. In these cases fever is often the only manifestation of an underlying serious infection; therefore, FN may be life-threatening, and these patients are candidates for inpatient management with IV broad-spectrum antibiotic therapy covering gram-negative pathogens [2–5]. All these precautions notwithstanding, the mortality for infections in hematological patients with neutropenia is still high [6].

Although the intuition to transfuse granulocytes from allogeneic donors in neutropenic patients dates back several decades [7], only the introduction of granulocyte-colony stimulating factor (G-CSF) as a mobilizing protocol has yielded adequate granulocyte apheresis products [8]. Nevertheless, despite the great number of studies conducted so far, it is still debated whether the transfusion of granulocyte products to treat or prevent life threatening infections results in a substantial survival increase [9–11]. Similarly, the recently concluded randomized controlled trial “Safety and Effectiveness of Granulocyte Transfusions in Resolving Infection in People with Neutropenia” (RING Study) failed to prove a true beneficial effect of granulocyte transfusions (GTs) [12].

Supportive care with GTs has been implemented in our center several years ago [13]. Over the years, however, PMN collection procedures have been standardized and clinical indications to GT therapy have been defined. In this study we revised the data relative to a large series of hematological patients consecutively treated with GTs in our department during FN episodes. It is generally acknowledged that at least 1-2x10^10^ granulocytes per transfusion should be given to elicit a therapeutic effect [14]. Therefore, provided that GTs significantly improve the infection outcome, patients receiving highest amounts of granulocytes should also maximally benefit from transfusions. Nevertheless, our initial results rapidly disproved our hypothesis, since patients surviving infections were receiving lesser amounts of polymorphonuclear cells (PMNs) than others. The European guidelines to the preparation, use and quality assurance of blood components recommend as standard dose of granulocyte apheresis products for adult patients 1.5–3.0x10^8^ cells/Kg of the recipient’s body weight [15]. We therefore divided our patients in three groups according to the median dose received during the infectious episode (IE), i.e. lower, equivalent or greater than 1.5–3.0x10^8^cells/Kg. Our results clearly show that different GT doses exert diverging effects on the infection outcome of hematological patients, suggesting that GTs can constitute a valuable tool to improve the outcome of infections in neutropenic patients, provided that adequate recipient-tailored doses are supplied.

## Materials and Methods

### Study design

We retrospectively analyzed the data of patients receiving at least one GT between January 2009 and December 2015 at our Hematology Department. Over the entire study period, the eligibility criteria for GTs were fever, an absolute neutrophil count (ANC) <500 cells/μL, evidence of fungal or bacterial infection (i.e., clinical signs of infection, positive cultures or biopsies, and radiological evidence) and unresponsiveness to the appropriate antimicrobial therapy for at least 48 hours. For all patients, the concomitant antibiotic and antifungal therapies were set according to the center guidelines. The primary outcome was the infection-related mortality (IRM) rate, defined as death from infection within 30 days after the last GT. In a subsequent analysis, the requirement of admission to the Intensive Care Unit (ICU) was also evaluated. All patient records were anonymized and de-identified prior the analysis. The study was approved by the Institutional Committees of the Catholic University, Faculty of Medicine (P/145/CE/2012) and registered at www.clinicaltrials.gov as NCT022544230.

### Data collection

Data were gathered by revising patient and donor clinical records and from electronic databases in use at our hospital (“Sistema Informativo Policlinico Gemelli” and “Emonet”), and were then entered in a Microsoft Excel database. For granulocyte donors, age, sex, date and number of granulocyte donations were recorded. For all patients included in the study, various clinical and laboratory data were retrieved. The following variables were included in the analysis: calendar year of transfusion, age, age >60 years, sex, underlying disease, chemotherapy line, allogeneic hematopoietic stem cell transplantation (allo-HSCT), days with ANC <500/μL, number of days of antimicrobial therapy before the first GT, G-CSF administration, blood stream infection, localized infection, fever of unknown origin (FUO), extensively drug resistant (XDR)-infection, pneumonia, bacterial infection, fungal infection, mixed bacterial and fungal infection, mono-microbial or poly-microbial infection, infection due to *Pseudomonas aeruginosa*, *Klebsiella pneumoniae*, *Aspergillus s*pecies and *Candida* species, number of transfusions, GT number/days with ANC <500/μL, overall amount of PMNs received and median dose of PMN per IE, evaluated both as continuous and categorical variable (i.e., low-dose <1.5x10^8^/Kg; standard-dose 1.5–3.0x10^8^/Kg and high-dose >3.0x10^8^/Kg; in selected analyses, patients in the low-dose and high-dose groups were combined and were overall evaluated).

### Cytokine profile evaluation

The cytokine profile was evaluated in plasma samples collected from 4 patients immediately before and 6–8 hours after GTs (n = 8) and in 6 hematological patients with FN receiving packed red blood cells (RBCs, n = 6), as control. Interleukin (IL)1β, IL6, Tumor Necrosis Factor-α (TNF-α) Interferon-γ (INF- γ) and monocyte chemotactic protein1 (MCP1) were dosed using Bio-Plex Cytokine Assays and Bio-Plex MAGPIX^TM^ Multiple Reader (Biorad). All experiments were performed in duplicate.

### Statistical analysis

Continuous variables were expressed as median (ranges) or mean (±SEM) and categorical variables as n (%). To compare continuous variables we used the Mann-Whitney U test, and for categorical variables we used the Fisher's exact test or the χ2 test, as appropriate. Relationship between variables was investigated by linear regression analysis.

Multivariate analysis was performed using a logistic regression model combining all variables with a p value <0.05 at univariate analysis with factors with acknowledged effect on the outcome of FN in hematological patients. In order to substantiate our findings, we repeated the analysis on additional models, either incorporating all variables evaluated in univariate analysis, or including only with significant p values at univariate analysis. The results were expressed as an odds ratio (OR) with a relative 95% confidence interval (95% CI).

The survival analysis was performed using the Kaplan–Meier method comparing differences through the log-rank test, expressing the results as a hazard ratio (HR), 95% CI.

A p value <0.05 was considered statistically significant. Analyses were performed using Graph Pad and IBM SPSS Statistics 21.0 software.

## Results

During the study period, 491 GTs were transfused to 96 patients during 114 IEs, with 18 patients suffering from more than one IE. Exhaustive data on donor characteristics and granulocyte apheresis products are shown in S1 Table. The first GT was given after a median number of 5 days of antimicrobial therapy (range 2–33); on average, 4 GTs (1–14) per patient were transfused. The median granulocyte dose per transfusion was 2.1x10^8^/Kg (0.4–7.3) and the median dose per IE was 8.9x10^8^/Kg (0.53–53.23) (Table 1). In all cases apheresis products were irradiated. Patient characteristics are summarized in Table 1.

**Table 1:** Patient and granulocyte transfusion characteristics in 114 infective episodes.

Characteristics	
**Age (years, median value range)**	46 (20–74)
**Male/Female**	74/40
**Underlying disease (n, %)**	
**Acute myeloid leukemia**	88 (77.2)
**Lymphoma**	15 (13.2)
**Acute lymphoblastic leukemia**	7 (6.2)
**Myelodysplastic syndromes**	2 (1.8)
**Multiple myeloma**	1 (0.8)
**Chronic lymphocytic leukemia**	1 (0.8
**Chemotherapy line (n, %**	
**First line**	73 (64.1)
**Subsequent lines**	41 (35.9)
**Duration of neutropenia (days, median value, range)**	18 (3–79)
**Antimicrobial therapy at first GT (days, median value, range)**	5 (2–33)
**ICU admission (n, %)**	
**Yes**	27 (23.6)
**No**	87 (76.4)
**Site of infection (n, %)**	
**Blood stream**	69 (60.5)
**Lung**	34 (29.8)
**Bowel**	5 (4.3)
**Others**	9 (7.9)
**Multiples (≥3 involved sites)**^a^	5 (4.3
**Allo-HSCT (n, %)**	
**Yes**	23 (20.1)
**No**	91 (79.9
**G-CSF treatment (n, %)**	
**Yes**	63 (55.2)
**No**	51 (44.8)
**Infection-related mortality (n, %)**	35 (30.7)
**Courses per patient (number of patients)**	
	1 (82)
	2 (11)
	3 (2)
	4 (1)
**Transfusions per course (median value, range)**	4 (1–14)
**PMN x 10**^**8**^**/kg/course (median value, range)**	8.90 (0.53–53.23)
**PMN x 10**^**8**^**/kg/transfusion (median value, range)**	2.16 (0.46–7.34)

GT: granulocyte transfusion, ICU: Intensive Care Unit; allo-HSCT: allogeneic-hematopoietic stem cell transplantation.

^a^ Four urinary, three soft tissues, and two ocular infections.

Overall, we recorded 35 deaths due to infections, with an overall IRM of 30.7%. The IRM rate fluctuated over the observation period, with no significant differences among years (p = 0.324). Detailed microbiological data are shown in Table 2.

**Table 2:** Microbiological records gathered in 114 infective episodes treated with granulocyte transfusions.

Infection	Number of cases
**Bacteria**	
**Klebsiella pneumoniae**	**40**
**Escherichia coli**	**18**
**Pseudomonas aeruginosa**	**13**
**Enterococcus faecium**	**13**
**Streptococcus oralis**	**4**
**Acinetobacter species**	**5**
**Streptococcus α-haemolyticus**	**5**
**Staphylococcus aureus**	**3**
**Others**^a^	**6**
**Fungi**	
**Aspergillus species**	**20**
**Candida species**	**22**
**Blastoschizomyces species**	**2**
**FUO**	**10**
**Monomicrobial infection**	**63**
**Polymicrobial infection**^b^	**41**
**XDR infection**	**38**

FUO: fever of unknown origin; XDR: extensively drug resistant

^*a*^*Micrococcus luteus* (N = 2); *Rothia mucilaginosa* (N = 1); *Corynebacterium amycolatum* (N = 1); *Legionella pneumophila* (N = 1); *Bacteroides ovatus* (N = 1).

^b^Gram-negative bacterial/fungal infections: N = 19; Gram-negative/Gram-positive bacterial infections: N = 16; Gram-negative/Gram-negative bacterial infections: N = 5; Gram-positive/Gram-positive bacterial infection: N = 1.

### IRM is significantly influenced by the median dose of PMN delivered during infection

The effect exerted by clinical, microbiological and transfusion-related variables on IRM at univariate analysis is shown in Table 3.

**Table 3:** Effect of clinical, microbiological and transfusion findings on requirement of ICU admission and infection-related mortality (univariate analysis).

Characteristics	Number of patients	IRM (%)	p	ICU (%)	p
**Sex**	Males: 74	23 (31.0)		18 (24.3)	
	Females: 40	12 (30.0)	>0.99	8 (20.0)	0.848
**Underlying disease**	Myeloid neoplasms: 9	32 (33.3)		24 (25.0)	
	Lymphoid neoplasms: 18	3 (16.6)	0.263	2 (11.1)	0.352
**Age over 60 years**^a^	Yes: 25	9 (36)		6 (30.0)	
	No: 89	26 (29.2)	0.459	20 (22.0)	0.788
**Chemotherapy lines**	First line: 73	26 (35.6)		19 (26.0)	
	Subsequent lines: 41	9 (21.9)	0.144	7 (17.0)	0.113
**Allo-HSCT**	Yes: 23	8 (34.7)		4 (17.3)	
	No: 91	27 (29.6)	0.622	12 (13.1)	0.587
**Blood stream infection**	Yes: 73	28 (38.3)		20 (27.3)	
	No: 41	7 (17)	**0.021**	6 (14.6)	0.168
**Bacterial infection**	Yes: 57	20 (35)		18(31.5)	
	No: 57	15 (26.3)	0.417	22 (17.5)	**0.043**
**Fungal infection**	Yes: 24	5 (20.8)		4 (16.6)	
	No: 90	30 (33.3)	0.321	22 (24.4)	0.586
**Polymicrobial infection**	Yes: 41	16 (39.0)		11 (26.8)	
	No: 73	19 (26.0)	0.204	15 (20.5)	0.490
**XDR infection**^b^	Yes: 38	18 (47.3)		11 (28.9)	
	No: 76	17 (22.3)	**0.009**	15 (19.7)	0.344
**Median PMN dose**^c^					
**<1.5**	18	8 (44.4)		5 (27.7)	
**1.5–3.0**	65	12 (18.5)		4 (6.1)	
**>3.0**	3	15 (48.4)	**0.004**	5 (16.1)	**0.035**
**Median PMN dose 1.5–3.0**^c^	Yes: 65	12 (18.4)		4 (6.1)	
	No: 76	23 (46.9)	**0.001**	10 (20.4)	**0.040**

ICU: Intensive Care Unit; Allo-HSCT: allogeneic-hematopoietic stem cell transplantation; XDR: extensively drug resistant.

^a^ Patients died for infection had a slightly higher median age than others (51 years, range 22–71 versus 45 years range 20–74, respectively, p = 0.055).

^b^XDR infections consisted of *Klebsiella pneumoniae* (N = 30), *Pseudomonas aeruginosa* (N = 7) and *Acinetobacter* (N = 5); 4 patients had multiple XDR infections.

^c^PMN doses are intended as x10^8^/Kg. Only GTs given before ICU admission were included in the analysis of the risk for ICU admission.

**Fig 1:**
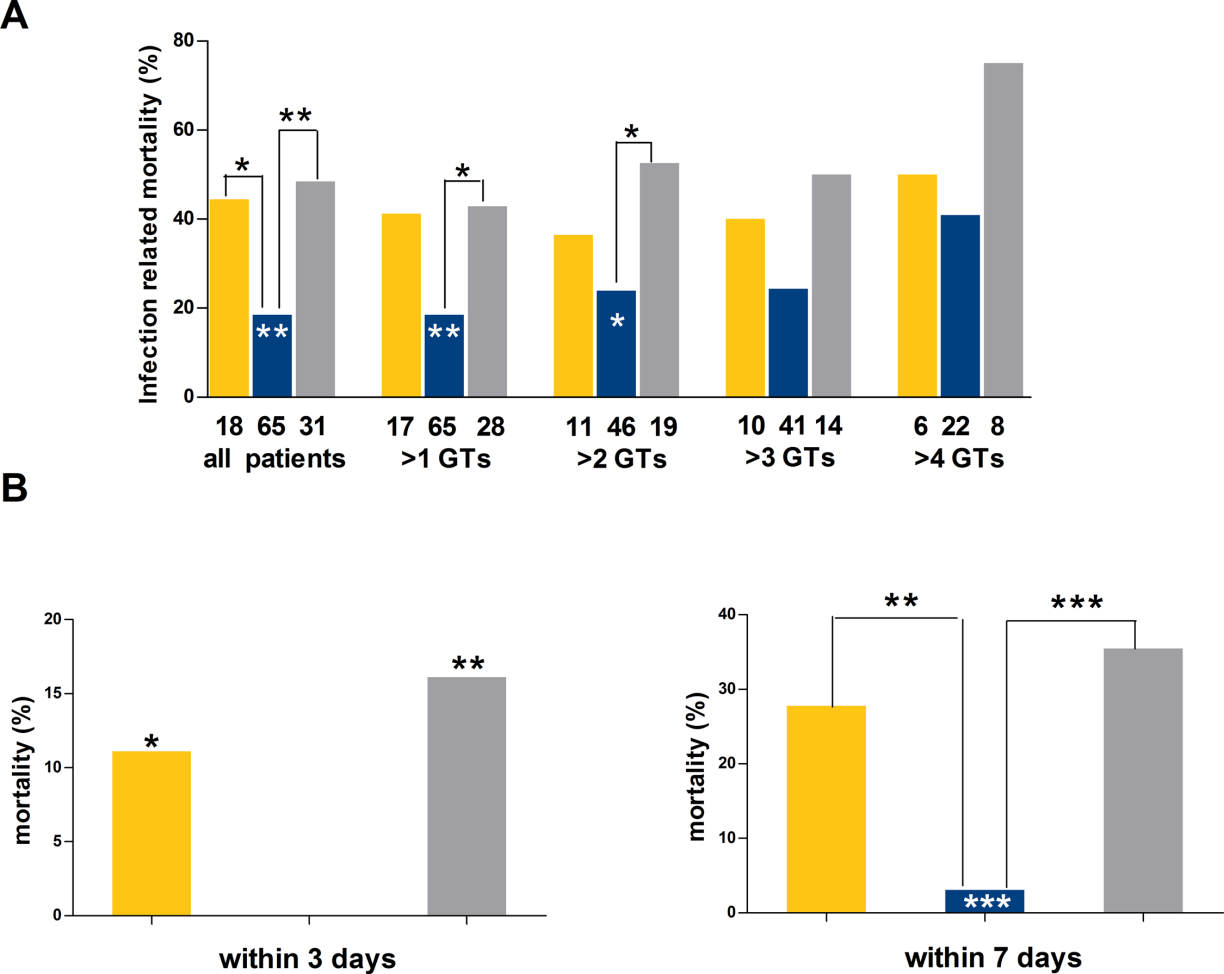
Infection-related mortality in patient grouped according to the median dose of granulocyte received. A) Mortality rate according to the number of granulocyte transfusions received. B) Mortality rate according to the time elapsed from the first granulocyte transfusion. Yellow bars represent patients in the low-dose group (median PMN dose inferior to1.5x10^8^/Kg), blue bars represent patients in the standard-dose group (median PMN dose 1.5–3.0x10^8^/Kg) and grey bars represent patients in the high-dose group (median PMN dose greater than 3.0x10^8^/Kg). White asterisks refer to standard-dose versus non-standard dose groups. *p<0.05; **p<0.001.

A higher IRM was detected in patients with blood stream infection (38.3% versus 17.0% in patients without blood stream infection, p = 0.021) and XDR infection (47.3% versus 22.3% in patients without XDR infection, p = 0.009) (Table 3). Except for a trend towards significance for higher age (51 years, range 22–71 in deceased patients versus 45 years, range 20–74 in surviving patients, p = 0.055) and infection due to *Klebsiella pneumoniae* (42.5% IRM rate versus 24.3% in patients with and without infection, respectively, p = 0.055), no further clinical or microbiological variables were associated with higher mortality (Table 3). The extent of antimicrobial therapy at the moment of the first GT was similar in surviving and deceased patients (5 days, range 2–33 in surviving patients and 6 days, range 2–33, in deceased patients, respectively, p = 0.065). Likewise, surviving and deceased patients received similar overall amount of PMNs during the IEs (8.2x10^8^/Kg, range 1.4–31.8, in surviving patients and 11.2x10^8^/Kg, range 0.5–53.2, in deceased patients, respectively). The IRM greatly varied according to the median PMN dose as follows: 18.4% in patients receiving 1.5–3.0x10^8^/Kg (i.e., standard dose), 44.4%, in patients receiving <1.5x10^8^/Kg (OR 3.5, 95% IC 1.1 to 10.8; p = 0.031 compared to the standard-dose group) and 48.4% in patients receiving >3.0x10^8^/Kg (OR 4.1, 95% IC 1.6 to10.6; p = 0.003 compared to the standard-dose group). Overall, patients in the low- and high-dose groups had higher IRM than others (OR 3.9; 95% IC 1.6 to 9.0; p = 0.001) (Table 3). These differences were further confirmed in 110 IEs where two or more GTs were given (Fig 1A). In particular, the IRM was 18.5% in the standard-dose group, 42.2% in patients receiving greater or lower PMN doses (OR 3.2; 95% IC 1.3 to 7.6, p = 0.009 in comparison with the standard-dose group) and 42.9% in the sole high-dose group (OR 3.3; 95% IC 1.2 to 8.7, p = 0.019 in comparison with the standard-dose group) (Fig 1). Similarly, among 76 patients receiving three or more GTs, the IRM was 23.9% in 46 patients in the standard-dose group and 46.6% in other patients (OR 2.7; 95% IC 1.0 to 7.4, p = 0.048), whereas among 19 patients in the sole high-dose group the IRM peaked at 52.6% (OR 3.5; 95% IC 1.1 to 10.9; p = 0.040 in comparison with the standard-dose group) (Fig 1A). The increased mortality among patients in the high- and low-dose groups was detectable since the first days after GTs (Fig 1B). Actually, of 7 patients deceased within 3 days after the first GT, 2 were in the low-dose group (11.1%) and 5 in the high-dose group (16.2%), whilst no deaths were observed in the standard-dose group (OR 19.8; 95% IC 0.9 to 433.9, p = 0.045 for low- versus standard-dose group and OR 27.1; 95% IC 1.4 to 506.9, p<0.002 for high- versus standard-dose dose group, respectively; Fig 1B). Similarly, of 18 patients deceased within one week after the first GT, 5 were in the low-dose group (27.1%), 11 in the high-dose group (35.4%), and only 2 in the standard-dose group (3.1%) (OR 12.1; 95% IC 2.1 to 69.4, p = 0.004 for low- versus standard-dose group and OR 17.3; 95% IC 3.5 to 84.8, p<0.001 for high- versus standard-dose dose group, respectively; Fig 1B).

In addition, patients in the high-dose and low-dose groups more frequently were admitted to ICU than those in the standard group (OR 3.9; 95% IC 1.1 to 13.3; p = 0.040) (Table 3). In order to assess possible worsening of pre-existing pulmonary infiltrates after infusion of large PMN amounts, chest X-rays performed within one week from the last GT in 34 patients with pneumonia were carefully revised. In 14 out of 34 patients, computed tomography scans were available as well. Worsening was defined as the enlargement/diffusion of pre-existing infiltrates, with or without increase of pleural effusions. Overall, worsening of pulmonary involvement was documented in 41% of patients with pneumonia: this finding was significantly associated with a higher mortality (p = 0.017) and more frequently occurred amid patients receiving high doses of granulocytes. In fact, 4 out of 7 patients in the high-dose group (57%) showed radiological worsening in comparison with 10 out of 27 patients (37%) receiving standard or low granulocyte doses. Although devoid of the any statistical validation for the low number of observations, these findings cast the doubt that transfusing high amounts of granulocytes might exacerbate pre-existing lung dysfunctions. Clinical and laboratory characteristics of patients divided according the dose received are shown in Table 4.

**Table 4:** Characteristics of patients grouped according to median PMN doses.

Characteristics	Low doses n = 18	Standard doses n = 65	High doses n = 31	p^a^	p^b^
**Males/females**	10/8	45/20	19/12	0.496	0.323
**Myeloid/lymphoid neoplasms**	16/2	52/13	29/2	0.194	0.111
**Age over 60 years (n,%)**	4 (22)	12 (18)	8 (25)	0.705	0.491
**First line therapy/Following line therapy**	16/2	39/26	18/13	0.056	0.330
**Allo-HSCT (n,%)**	0 (0)	13 (20.0)	10 (32.2)	**0.025**	>0.99
**Blood stream infection (n,%)**	11 (61.1)	28 (43.0)	18 (58.0)	0.230	0.079
**FUO/Monomicrobial/polymicrobial infection (n)**	3/8/7	7/36/22	0/19/12	0.863	0.694
**Bacterial infection (n, %)**	11 (61.1)	28 (43.0)	18 (58.0)	0.230	0.130
**Fungal infection (n, %)**	2 (11.1)	16 (24.6)	6 (19.3)	0.445	0.356
***Klebsiella pneumoniae* infection (n,%)**	8 (44.4)	21 (32.3)	11 (35.4)	0.633	0.553
**XDR infection (n, %)**	7 (38.8)	20 (30.7)	11 (35.4)	0.776	0.551
**G-CSF treatment (n, %)**	8 (44.4)	38 (58.4)	17 (54.8)	0.452	0.570
**Antimicrobial therapy at first GT (days, median value, range)**	5 (2–14)	5 (2–33)	5.5 (2–33)	0.550	0.604
**IRM (n,%)**	8 (44.4)	12 (18.5)	15 (48.4)	**0.005**	**0.002**
**Age (years, median value, range)**	53 (38–74)	45 (20–71)	49 (21–74)	0.107	0.087
**Days of neutropenia, median value (range)**	18.5 (10–79)	20 (6–76)	17 (3–73)	0.700	0.470
**Transfusions per course (median value, range)**	1 (1–5)	3 (1–10)	2 (1–11)	0.200	0.109

Low doses are intended as <1.5x10^8^/Kg; standard doses are intended as 1.5-3x10^8^/Kg and high doses are intended as >3x10^8^/Kg. ICU: intensive care unit; allo-HSCT: allogeneic haematopoietic stem cell transplantation; FUO: fever of unknown origin; XDR: extensively drug resistant; GT: granulocyte transfusion; IRM: infection-related mortality.

^a^comparison among three groups.

^b^comparison between standard dose-group and cumulated low dose- and high dose-groups.

We then combined in a multiple logistic regression model all variables with a significant impact on IRM at univariate analysis (median PMN dose categorized as standard or not, blood stream infection and XDR infection), together with age >60 years and allo-HSCT, two conditions whose negative impact on the outcome of hematological patients with FN is widely recognized [1–6]. In our analysis, to receive a median dose lower than 1.5x10^8^/Kg or greater than 3.0x10^8^/Kg was the only independent factor significantly associated with higher mortality (OR 3.7, 95% IC 1.5 to 8.9, p = 0.004) (Table 5). Similar results were obtained after excluding from the analysis age >60 years and allo-HSCT (OR 3.7, 95% IC 1.5 to 9.1, p = 0.003) or after including all the variables evaluated in univariate analysis (OR 3.8, 95% IC 1.5 to 9.4, p = 0.004).

**Table 5:** Combined effect of clinical and transfusion parameters on infection-related mortality and survival estimates

	OR (95% CI)	p
**Age > 60 years**	1.4 (0.5–4.2)	0.482
**allo-HSCT**	1.0 (0.3–3.0)	0.938
**Blood stream infection**	1.8 (0.5–5.6)	0.295
**XDR infection**	2.4 (0.9–6.6)	0.069
**Median PMN dose**^a^	3.7 (1.5–8.9)	0.004

allo-HSCT: allogeneic-hematopoietic stem cell transplantation; XDR: extensively drug resistant.

^a^Median PMN doses are intended as standard (1.5–3.0 x10^8^/Kg) or not (<1.5x10^8^/Kg and > 3.0x10^8^/Kg).

**Fig 2:**
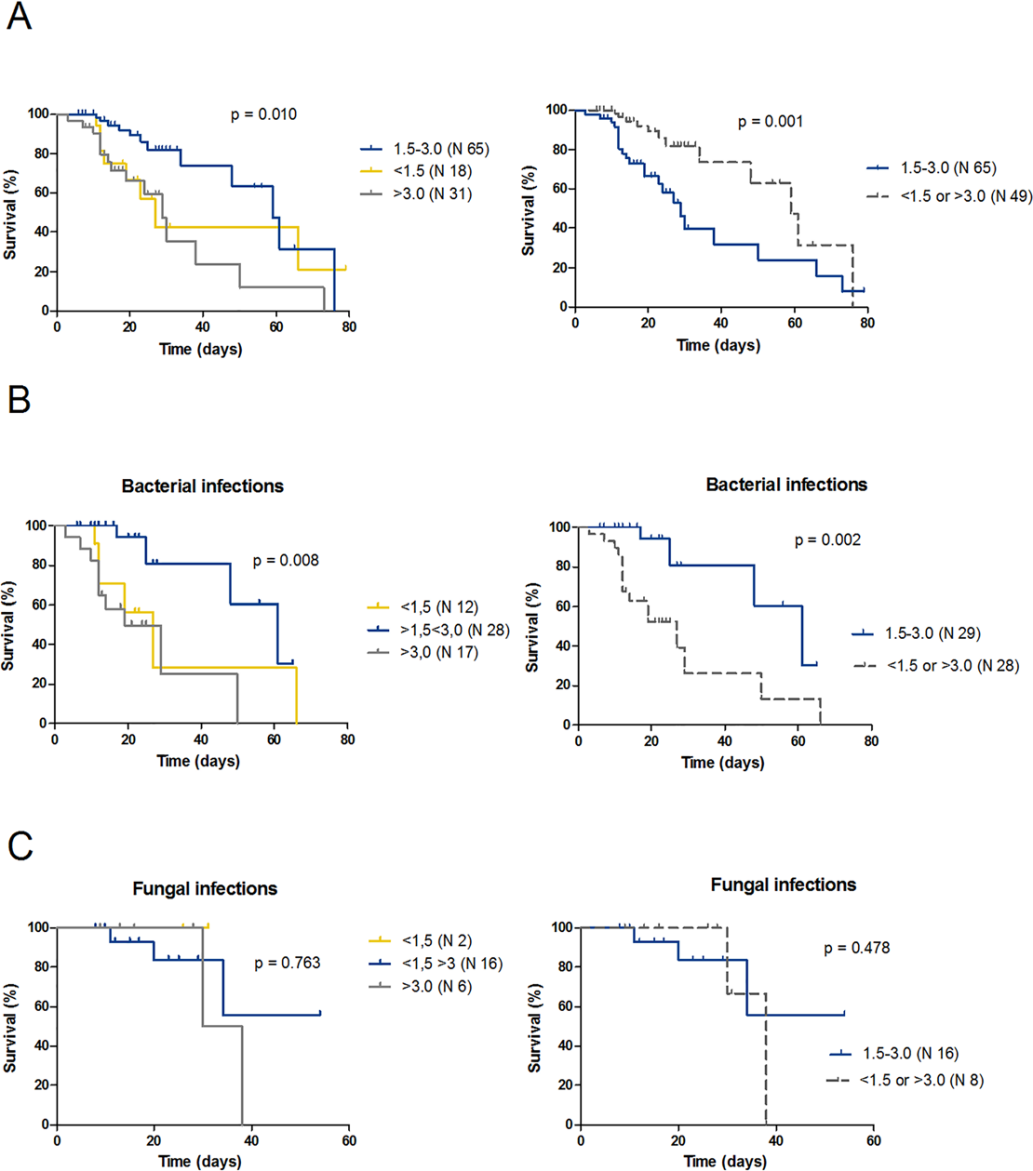
Survival curves according to the median doses of granulocytes received. The survival was determined as the interval between the first day with ANC<500/μL and death or neutropenia recovery. Differences were estimated using the log-rank test. **A)** Survival of neutropenic patients during overall 114 infectious episodes. **B)** Survival of neutropenic patients during bacterial infections **C)** Survival of neutropenic patients during disseminated fungal infections. Yellow curves represent patients in the low-dose group (median PMN dose inferior to1.5x10^8^/Kg), blue curves represent patients in the standard-dose group (median PMN dose 1.5–3.0x10^8^/Kg) and grey curves represent patients in the high-dose group (median PMN dose greater than 3.0x10^8^/Kg), dotted curves represent cumulated patients in low and high dose groups.

Fig 2A illustrates the survival of neutropenic patients, measured as the interval between the first day with ANC<500/μL and death or neutropenia recovery. The median survival significantly differed according to the PMN dose received (29 days versus 59 days, in standard- and nonstandard-groups, respectively; HR 2.6, 95% IC 1.3 to 5.3; p = 0.001).

### The dose-dependent effect of GTs is detected in bacterial but not fungal infections

In order to understand if GTs were similarly effective against bacterial and fungal infections, we selected from the entire cohort of patients two subgroups with bacterial or fungal infections, and we investigated the effect on IRM of the same variables that had been tested on the whole cohort of patients. Overall, 57 patients had bacterial infections (12 in the low-dose group, 28 in the standard-dose group and 17 in the high-dose group) and 24 had disseminated fungal infections (2 in the low-dose group, 16 in the standard-dose group and 6 in the high-dose group). Results are shown in S2 Table. Regarding bacterial infections, receiving median PMN doses lower than 1.5 or greater than 3.0x10^8^/Kg was associated with higher mortality both at univariate and multivariate analysis (OR 18.4, 95% CI 2.9 to 114.6, p = 0.002, S2 Table). In contrast, among patients with invasive fungal infections (18 *Aspergillus* species, 5 *Candida* species and 1 *Blastoschizomyces* species), we failed to identify any association between IRM and the investigated variables, including the median dose of PMN received (S2 Table). Moreover, in patients with bacterial infections, median survivals significantly differed in according to different PMN doses received (27 days versus 61 days, in standard- and nonstandard-groups, respectively; HR 4.0, 95% IC 1.6 to 10.1; p = 0.002) (Fig 2B), whilst no dose-dependent effect of GTs was observed among neutropenic patients with fungal infection (Fig 2C).

### Pro-inflammatory cytokine response is boosted after GTs

**Fig 3:**
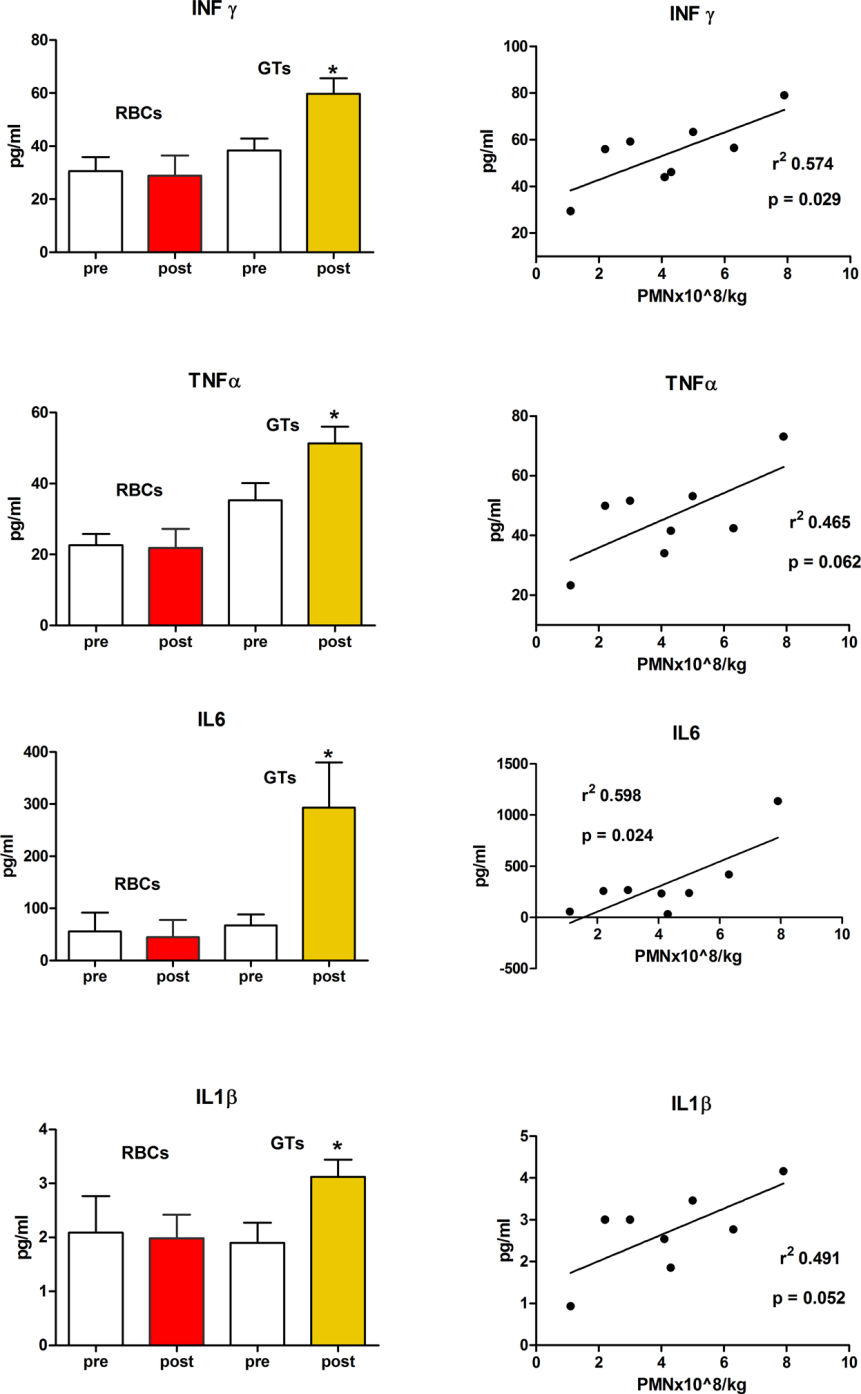
Cytokine plasma concentrations before and after transfusions. IL1β,IL6, TNF-α and INF-γ levels were measured in 4 patients receiving 8 GTs and in 6 patients receiving RBCs transfusions, as control. In all patients, samples were collected immediately before and 6–8 hours after transfusions. Results are expressed as mean values±SEM. The cytokine concentration observed in samples collected post-GTs were proportional to the PMN dose delivered. *p<0.05

Although the poor outcome of patients in the low-dose group could result from the trivial effect of GTs against infection, the high IRM among patients receiving larger amounts of granulocytes suggested that GTs could elicit additional and potentially detrimental responses. Therefore, we evaluated if transfused granulocytes were able to induce in the recipients the abnormal increase of cytokines and chemokines implicated in the pathophysiology of sepsis [16]. Four patients receiving GTs were evaluated, and, as control, the same measures were carried out in further 6 FN patients receiving RBCs transfusions. Results are illustrated in Fig 3. A total of 8 GTs were given to 4 patients, with a median amount of 4.1x10^8^ cells/Kg (range 1.1 to 7.9x10^8^/Kg) per transfusion. A significant release of IL1β,IL6, TNF-α and INF-γ (but not MCP-1, data not shown) was observed, whereas no considerable increase occurred after RBCs transfusions. In all cases, cytokine increase was proportional to the PMN dose delivered (Fig 3).

## Discussion

Transfusion therapy for anemia or thrombocytopenia is tailored to elicit a recognizable clinical improvement in recipients [17]. In neutropenic patients, however, GTs have been demonstrated to increase the WBC count [9–11] but strong evidence of a decrease in mortality due to infections in randomized trials is still lacking [12,18–21].

It is generally believed that patients attaining maximal benefits from GTs are those receiving the highest amounts of PMN. In our analysis, we considered as “standard” the dose of 1.5–3.0x10^8^ PMN/Kg [15] and we accordingly stratified patients into low-, standard- and high-dose groups. Indeed, we observed that patients receiving median doses of 1.5–3.0x10^8^ PMN/kg had a reduced IRM and lower ICU admission rate in comparison with patients receiving both lower or higher doses of granulocytes.

Previous studies suggested an increased infection control in patients receiving GTs at doses greater than 10x10^9^ cells [10,11]. Recently, the RING Study demonstrated that very high doses of granulocytes (i.e. >6x10^8^/kg) elicit better responses than lower doses (i.e. <6x10^8^/kg) [12]. Only two patients in our series received more than 6x10^8^/kg granulocytes, and in both of them only one GT was given, so that no comparison between studies is sensitive. Nevertheless, a greater number of patients in the RING series suffered from disseminated fungal infections in comparison with our cohort (46% versus 21%, respectively). Notably, we failed to demonstrate any dose-dependent GT effect in patients with disseminated fungal infections. Our conclusions are drawn from a low number of patients, and undoubtedly they ought to be carefully interpreted. Nevertheless, it might be conceivable that fungal infections may necessitate very high doses of PMN, whilst lower doses, ranging from 1.5 and 3x10^8^/Kg, are sufficient to overcome bacterial infections. On the other hand, it is noteworthy that in pediatric setting, where the majority of patients usually receive large amount of PMN due to the low body weight, the benefit of high dose-GTs has not yet been validated [22].

Although our analysis explored one of the largest series of patients so far treated in a single center, the lack of a control group precludes adjusting for possible confounding variables, which could have biased our observations. In addition, the retrospective nature of the study requires great caution in interpreting the results. Notwithstanding these limitations, the poorer outcome of patients transfused with low doses of PMN suggest that GTs given at adequate amounts might results in a reliable advantage for recipients. Nevertheless, to explain the increased IRM among patients in the high-dose group is quite challenging. It could be speculated that apheresis products with larger PMN amounts might have been assigned to sicker patients. However, it is evident that the quality of the apheresis products does only depends on the mobilization performance of donors (S1 Table), so that possible alternative explanations deserve to be considered.

The occurrence of adverse events as a consequence of GTs has been previously reported [23]. Side effects mainly consist of pulmonary complications in patients with pre-existing pneumonia, and they are rarely life-threatening [14]. In our patients no GT-related serious adverse events were recorded, nor pulmonary involvement was more frequent among deceased patients. Nevertheless, we noticed that a significant proportion of patients with pneumonia showed worsening of radiological findings during GTs, suggesting that respiratory function and radiological findings should be carefully monitored during GTs. Importantly, in this study we focused on the median granulocyte dose, which reflects the whole GT treatment rather than an individual transfusions. Indeed, we cannot ascribe the increased IRM observed among patients in the high-dose group to eventual single transfusion toxicity, but additional and possibly repeated effects of GTs in the recipients might be involved.

Red cell transfusions are independent predictors of morbidity and mortality in critically ill patients, and residual leukocytes in blood cell products are important contributors to the detrimental effects of transfusions [24,25]. The use of leukoreduced red cell products may reduce mortality and morbidity, including decrease of acute kidney injury, acute respiratory distress syndrome, transfusion related acute lung injury and transfusion-associated circulatory overload [25]. In addition to these acute complications, blood products can cause profound negative effects on the human immune system, a condition termed transfusion-related immune modulation (TRIM) [25]. Mechanisms for TRIM include suppression of cytotoxic cell and monocyte activity, release of immunosuppressive prostaglandins, inhibition of interleukin-2 production, and increase in suppressor T cell activity [25–27]. To our knowledge, no data are available regarding an eventual TRIM effect of GTs. However, neutrophils are an important source of mediators of the systemic inflammatory response as well as of inhibitors of the immune system [16,28,29]. Our preliminary data suggest that following GTs a dose-dependent increase occurs of several mediators of the immune response, both with pro-inflammatory properties, such as IL1β, IL6 and TNF-α or immunoregulatory activity, such as INF-γ. It is widely acknowledged that an unbalanced inflammatory response is implicated in the pathophysiology of sepsis [16,29] as well as of pulmonary transfusion reactions [30]. Studies in trauma patients show that multi-organ failure develops after overwhelming severe inflammatory response syndrome (SIRS) of the innate immune system, not balanced by compensatory anti-inflammatory response from the adaptive immunity system [31,32]. More recently, transcriptome study of circulating leukocytes in patients with severe trauma, demonstrated a "genomic storm" affecting almost all cellular functions and pathways [33]. This response was consistent with simultaneously increased expression of genes involved in the systemic inflammatory, innate immune, and compensatory anti-inflammatory responses, as well as in the suppression of genes involved in adaptive immunity [33]. An analogous response was observed in healthy subjects receiving low-dose bacterial endotoxin [33]. Interestingly, the longer duration of this genomic reprioritization was associated with delayed recovery and death [33]. Indeed, we could speculate that transfusing high amounts of granulocytes in the “septic milieu” of our patients might mimic either repeated inflammatory stimuli or, in alternative, they may amplify and prolong the primary inflammatory response.

## Conclusions

On the whole, our observations offer a new perspective to assess the efficacy of GTs and suggest that they may fuel a wide range of immunological effects which may variably affect IRM. Future studies exploring the biology related to GTs are recommended, to definitely decipher the actual benefits of this therapeutic approach.

## Supporting Information

S1 Table Characteristics of donors and apheresis products. Donations were collected from 259 volunteers enrolled among patient’s friends or relatives not eligible for stem cell donation: 232 donors underwent two consecutive apheresis procedures, and 27 donors performed only one donation. Apheresis procedures were performed 12 and 36 hours after a single G-CSF administration (300 μg), using a continuous flow separator (COBE Spectra, Terumo BCT, Lakewood, CO, USA). In cases of ABO and Rh(D) blood group incompatibility (219 on 491 products; 44.5%), granulocyte concentrates were subjected to post-collection red blood cell removal by sedimentation with succinylgelatin (Eufusin, Medacta Italia, Milan, Italy), achieving a total RBC volume lower than 30 mL per transfusion. Overall, 153 units (31%) contained less than 10x10^9^ granulocytes. In all cases apheresis products were irradiated. §doses are intended before red blood cell removal.
(DOCX)

S2 Table Clinical and transfusion findings in 57 bacterial infections and 24 disseminated fungal infections. On the whole, 20 deaths were recorded among bacterial infections and 5 among fungal infections. IRM: infection-related mortality; ICU: Innsive care Unit; Allo-HSCT: allogeneic hematopoietic stem cell transplantation; XDR: extensively drug resistant; GTs: granulocyte transfusions.
(DOCX)
