# Supplementary material for: Dose-Dependent Effect of Granulocyte Transfusions in Hematological Patients with Febrile Neutropenia
Source: PLoS One. 2016 Aug 3;11(8):e0159569. doi: 10.1371/journal.pone.0159569 (PMC4972400; doi:10.1371/journal.pone.0159569)
Supplement: S1 Table — Donations were collected from 259 volunteers enrolled among patient’s friends or relatives not eligible for stem cell donation: 232 donors underwent two consecutive apheresis procedures, and 27 donors performed only one donation. Apheresis procedures were performed 12 and 36 hours after a single G-CSF administration (300 μg), using a continuous flow separator (COBE Spectra, Terumo BCT, Lakewood, CO, USA). In cases of ABO and Rh(D) blood group incompatibility (219 on 491 products; 44.5%), granulocyte concentrates were subjected to post-collection red blood cell removal by sedimentation with succinylgelatin (Eufusin, Medacta Italia, Milan, Italy), achieving a total RBC volume lower than 30 mL per transfusion. Overall, 153 units (31%) contained less than 10x109 granulocytes. In all cases apheresis products were irradiated. §doses are intended before red blood cell removal. (DOCX) [file pone.0159569.s001.docx]

**Table 1S.** **Characteristics of donors and apheresis products.**

| **Donor characteristics** |  |  |
| --- | --- | --- |
| Total donors | 259 |  |
| Total numbers of PMN donations | 491 |  |
| Male/Female | 220/39 |  |
| Age (years, median value, range) | 40 (20-59) |  |
| Blood volume processed (ml, range) | 5178 (3423-6919) |  |
| WBC x10^9^/L at collection (median value, range)  1st donation  2nd donation | 25230 (8120-46750)  13955 (8120-33680) | *p*  < 0.0001 |
| Neutrophil x10^9^/L (median value, range)  1^st^ donation  2^nd^ donation | 21660 (6438-41421)  10924 (6098-28527) | < 0.0001 |
| Hemoglobin level (g/dL, median value, range)  1^st^ donation  2^nd^ donation | 14.8 (11.2-17.4)  14 (11.1-16.9) | < 0.0001 |
| Platelets x10^9^/L (median value, range)  1^st^ donation  2^nd^ donation | 236000 (123000-434000)  216000 (104000-541000) | < 0.0001 |
| **Apheresis product’ characteristics** |  |  |
| Duration of apheresis (minutes, median value range) | 175 (122-201) |  |
| Anticoagulant infused (ml, median value, range) | 540 (259-654) |  |
| Volume of cell products (ml, median value, range) | 487 (230-654) |  |
| ^§^PMN x10^9^ collected (median value, range)  1st donation  2nd donation | 15.2 (0.8-75.8)  26 (0.8-75.8)  10 (1.1-30.1) | < 0.0001 |
| RBC collected (ml, median value, range)  1st donation  2nd donation | 120 (21-249)  133 (21-249)  108 (31-231) | < 0.0001 |

Donations were collected from 259 volunteers enrolled among patient’s friends or relatives not eligible for stem cell donation: 232 donors underwent two consecutive apheresis procedures, and 27 donors performed only one donation. Apheresis procedures were performed 12 and 36 hours after a single G-CSF administration (300 μg), using a continuous flow separator (COBE Spectra, Terumo BCT, Lakewood, CO, USA). In cases of ABO and Rh(D) blood group incompatibility (219 on 491 products; 44.5%), granulocyte concentrates were subjected to post-collection red blood cell removal by sedimentation with succinylgelatin (Eufusin, Medacta Italia, Milan, Italy), achieving a total RBC volume lower than 30 mL per transfusion. Overall, 153 units (31%) contained less than 10x10^9^ granulocytes. In all cases apheresis products were irradiated. ^§^doses are intended before red blood cell removal.
