# Supplementary material for: Dose-Dependent Effect of Granulocyte Transfusions in Hematological Patients with Febrile Neutropenia
Source: PLoS One. 2016 Aug 3;11(8):e0159569. doi: 10.1371/journal.pone.0159569 (PMC4972400; doi:10.1371/journal.pone.0159569)
Supplement: S2 Table — On the whole, 20 deaths were recorded among bacterial infections and 5 among fungal infections. IRM: infection-related mortality; ICU: Innsive care Unit; Allo-HSCT: allogeneic hematopoietic stem cell transplantation; XDR: extensively drug resistant; GTs: granulocyte transfusions. (DOCX) [file pone.0159569.s002.docx]

**S2 Table. Clinical and transfusion findings in 57 bacterial infections and 24 disseminated fungal infections.**

| **Characteristics** | **Bacterial infections (N =57)** | **p*** | **p§** | **Fungal infections (N=24)** | **p** |
| --- | --- | --- | --- | --- | --- |
| Sex  Number of pts (IRM%) | Male: 39 (38.4)  Female: 18 (27.7) | 0.555 | 0.077 | Male: 9 (22.2)  Female: 15 (20.0) | >0.999 |
| Underlying disease  Number of pts (IRM%) | Myeloid neoplasms: 49 (36.7)  Lymphoid neoplasms: 8 (25.0) | 0.699 | 0.522 | Myeloid neoplasms: 19 (26.3)  Lymphoid neoplasms: 5 (0) | 0.544 |
| Age over 60 years  Number of pts(IRM%) | Yes: 12 (50.0)  No: 45 (31.1) | 0.309 | 0.101 | Yes: 3 (33.3)  No: 21 (19.0) | 0.521 |
| Chemotherapy lines  Number of pts (IRM%) | First line 38 (36.8)  Subsequent lines: 19 (31.5) | 0.775 | 0.966 | First line: 13 (30.7)  Subsequent lines: 11 (9.0) | 0.327 |
| ICU admission  Number of pts (IRM%) | Yes: 19 (78.9)  No: 38 (13.1) | **<0.001** | - | Yes: 4 (100)  No: 20 (5.0) | **<0.001** |
| Allo-HSCT  Number of pts (IRM%) | Yes: 15 (40.0)  No: 42 (33.3) | 0.755 | 0.512 | Yes: 4 (0)  No: 20 (25.0) | 0.544 |
| Blood stream infection  Number of pts (IRM%) | Yes: 54 (37.0)  No: 3 (0) | 0.545 | 0.999 | Yes: 3 (0)  No: 21 (23.8) | >0.999 |
| Pneumonia  Number of pts (IRM%) | Yes: 4 (50.0)  No: 53 (33.9) | 0.607 | 0.999 | Yes: 22 (22.7)  No: 2 (0) | >0.999 |
| XDR infection  Number of pts (IRM%) | Yes: 24 (41.6)  No: 33 (30.3) | 0.411 | 0.232 | Yes: 1 (0)  No: 23 (21.7) | >0.999 |
| Median PMN dose 1.5-3.0x10^8^/kg  Number of pts (IRM%) | Yes: 28 (14.2)  No: 29 (55.1) | **0.002** | **0.002** | Yes: 16 (18.7)  No: 8 (25.0) | >0.999 |
| Age, years,  median value (range) | Live: 44 (35-74)  Deaths: 55 (22-71) | 0.067 | 0.221 | Live: 45 (36-64)  Deaths: 49 (28-56) | 0.520 |
| Days of neutropenia,  median value (range) | Live: 17 (6-65)  Deaths: 15.5 (3-66) | 0.940 | 0.368 | Live: 20 (8-54)  Deaths: 30 (11-38) | 0.255 |
| GTs per course,  median value (range) | Live: 3 (2-6)  Deaths: 3.5 (1-14) | 0.390 | 0.558 | Live: 4 (2-12)  Deaths: 8 (3-14) | 0.121 |
| Number of transfusions/days of neutropenia, %median value (range) | Live: 16.6 (5.3-57.1)  Deaths: 20.8 (3.2-100) | 0.375 | 0.930 | Live: 25 (3.4-75.0)  Deaths: 40 (7.8-72.7) | 0.393 |

On the whole, 20 deaths were recorded among bacterial infections and 5 among fungal infections. IRM: infection-related mortality; ICU: Intensive care Unit; Allo-HSCT: allogeneic hematopoietic stem cell transplantation; XDR: extensively drug resistant; GTs: granulocyte transfusions. *univariate analysis; §multivariate analysis
